# The effect of single low-dose primaquine treatment for uncomplicated *Plasmodium falciparum* malaria on haemoglobin levels in Ethiopia: a longitudinal cohort study

**DOI:** 10.1186/s12936-024-05021-x

**Published:** 2024-07-12

**Authors:** Kassahun Habtamu, Hallelujah Getachew, Ashenafi Abossie, Assalif Demissew, Arega Tsegaye, Teshome Degefa, Xiaoming Wang, Ming-Chieh Lee, Guofa Zhou, Solomon Kibret, Christopher L. King, James W. Kazura, Beyene Petros, Delenasaw Yewhalaw, Guiyun Yan

**Affiliations:** 1 Department of Microbial, Cellular & Molecular Biology, Addis Ababa University, Addis Ababa, Ethiopia; 2 Department of Medical Laboratory Sciences, Menelik II Medical and Health Science College, Addis Ababa, Ethiopia; 3 Department of Medical Laboratory Sciences, Arbaminch College of Health Sciences, Arbaminch, Ethiopia; 4 Department of Medical Laboratory Sciences, College of Medicine and Health Sciences, Arba Minch University, Arbaminch, Ethiopia; 5 Department of Medical Laboratory Sciences, College of Medicine and Health Sciences, Ambo University, Ambo, Ethiopia; 6 College of Natural Science, Department of Biology, Jimma University, Jimma, Ethiopia; 7 School of Medical Laboratory Sciences, Faculty of Health Sciences, Jimma University, Jimma, Ethiopia; 8 Program in Public Health, University of California at Irvine, Irvine, CA 92697 USA; 9 West Valley Mosquito and Vector Control District, Ontario, CA USA; 10 Center for Global Health & Diseases, School of Medicine, Case Western Reserve University, Cleveland, 44106 OH USA; 11 Tropical and Infectious Diseases Research Center (TIDRC), Jimma University, Jimma, Ethiopia

**Keywords:** Plasmodium falciparum, Malaria elimination, Primaquine, Artemisinin-based combination therapies, Haemoglobin, G6PD deficiency, Ethiopia

## Abstract

**Background:**

To interrupt residual malaria transmission and achieve successful elimination of *Plasmodium falciparum* in low-transmission settings, the World Health Organization (WHO) recommends the administration of a single dose of 0.25 mg/kg (or 15 mg/kg for adults) primaquine (PQ) combined with artemisinin-based combination therapy (ACT), without glucose-6-phosphate dehydrogenase (G6PD) testing. However, due to the risk of haemolysis in patients with G6PD deficiency (G6PDd), PQ use is uncommon. Thus, this study aimed to assess the safety of a single low dose of PQ administered to patients with G6PD deficiency.

**Methods:**

An observational cohort study was conducted with patients treated for uncomplicated *P. falciparum* malaria with either single-dose PQ (0.25 mg/kg) (SLD PQ) + ACT or ACT alone. Microscopy-confirmed uncomplicated *P. falciparum* malaria patients visiting public health facilities in Arjo Didessa, Southwest Ethiopia, were enrolled in the study from September 2019 to November 2022. Patients with uncomplicated *P. falciparum* malaria were followed up for 28 days through clinical and laboratory diagnosis, such as measurements of G6PD levels and haemoglobin (Hb) concentrations. G6PD levels were measured by a quantiative CareSTART™ POCT S1 biosensor machine. Patient interviews were also conducted, and the type and frequency of clinical complaints were recorded. Hb data were taken on days (D) 7, 14, 21, and 28 following treatment with SLD-PQ + ACT or ACT alone.

**Results:**

A total of 249 patients with uncomplicated *P. falciparum* malaria were enrolled in this study. Of these, 83 (33.3%) patients received ACT alone, and 166 (66.7%) received ACT combined with SLD-PQ treatment. The median age of the patients was 20 (IQR 28–15) years. G6PD deficiency was found in 17 (6.8%) patients, 14 males and 3 females. There were 6 (7.2%) and 11 (6.6%) phenotypic G6PD-deficient patients in the ACT alone and ACT + SLD-PQ arms, respectively. The mean Hb levels in patients treated with ACT + SLD-PQ were reduced by an average of 0.45 g/dl (95% CI = 0.39 to 0.52) in the posttreatment phase (D7) compared to a reduction of 0.30 g/dl (95% CI = 0.14 to − 0.47) in patients treated with ACT alone (*P* = 0.157). A greater mean Hb reduction was observed on day 7 in the G6PDd ACT + SLD-PQ group (− 0.60 g/dL) than in the G6PDd ACT alone group (− 0.48 g/dL); however, there was no statistically significant difference (*P* = 0.465). Overall, D14 losses were 0.10 g/dl (95% CI = − 0.00 to 0.20) and 0.05 g/dl (95% CI = − 0.123 to 0.22) in patients with and without SLD-PQ, respectively (*P* = 0.412).

**Conclusions:**

This study’s findings indicate that using SLD-PQ in combination with ACT is safe for uncomplicated *P. falciparum* malaria regardless of the patient's G6PD status in Ethiopian settings. Caution should be taken in extrapolating this finding in other settings with diverse G6DP phenotypes.

## Background

The global decline in malaria incidence due to increased malaria control measures, particularly the use of artemisinin-based combination therapy (ACT), has stimulated efforts to eliminate *Plasmodium falciparum* and sparked an upsurge in interest in therapies to stop transmission [1]. In many malaria-endemic areas, artemisinin derivatives are very effective against both asexual and young *P. falciparum* gametocytes, and this has considerably enhanced the global reduction in malaria transmission [2]. However, mature gametocytes continue to exist after ACT at the microscopic or submicroscopic level, and residual transmission is not interrupted after ACT [3, 4]. This is the reason why artemisinin derivatives against mature *P. falciparum* gametocytes are not effective in preventing transmission [5].

To treat *P. falciparum* malaria, the World Health Organization (WHO) recommends incorporating a single low dose of primaquine (SLD-PQ, 0.25 mg/kg of body weight) into artemisinin-based combination therapy (ACT), as part of pre-elimination or elimination programmes [6] and as part of artemisinin resistance containment programmes [7]. In low-endemic settings, the combination of this gametocytocidal drug with ACT is effective [8, 9], and this combination may significantly reduce malaria transmission [10]. While PQ is a promising treatment for relapse of *Plasmodium ovale* and *Plasmodium vivax*, cautions about its safety should not be overlooked, particularly in patients with glucose-6-phosphate dehydrogenase deficiency (G6PDd) [11]. Compared to single low-dose (SLD) PQ treatment, the dosage regimen used is noticeably higher, which could put G6PDd patients at greater risk of haemolysis [12]. The WHO currently recommends adding a single dose of 0.25 mg/kg to ACT without glucose-6-phosphate dehydrogenase (G6PD) to reduce transmission in low-transmission settings and accelerate the elimination of *P. falciparum* [8, 13]. However, due to fears of haemolysis in people with G6PD deficiency (G6PDd) [14], the use of PQ is not as common as anticipated. Hence, the broad use of PQ is hampered by safety concerns of haemolysis in individuals with G6PDd, the most prevalent hereditary enzyme defect reported in all malaria-endemic areas [15].

An estimated 400 million people worldwide suffer from G6PDd, which is an X-linked genetic disorder in malaria-endemic countries [16]. The frequency of G6PDd varies significantly from region to region, even within a country, with the highest prevalence reported in Africa. Additionally, migration and resettlement have an impact on the spread of this genetic disorder [17, 18]. Due to safety concerns, especially for those with G6PDd, which affects up to 37.5% of the continent's population [19], the use of PQ is also restricted, particularly in Africa. In Africa, the predominant G6PD deficiency variants are the mild A-type, while in Asia, there is a heterogeneous mix, with the Mediterranean variant being the most severe. These variations underscore the complexity of managing G6PDd and the importance of tailored healthcare approaches based on regional prevalence and genetic diversity [20]. The safety of PQ in relation to haemolysis at the individual level has prevented its use despite the benefit being solely at the population level [21].

With a growing body of evidence on the safety of the administration of SLD-PQ in recent years, there have been indications that SLD-PQ is well tolerated in G6PDd patients [22].Several randomized and controlled clinical trials on the safety and efficacy of ACT versus SLD-PQ have been conducted in South Africa [21], Tanzania [23, 24], Senegal [6], Swaziland [25], Burkina Faso [26], and Uganda [27], supporting the use of the WHO-recommended SLD-PQ without G6PD testing during the preelimination and elimination phases of malaria.

Although previous studies have shown that SLD-PQ is safe even for people with G6PDd, the WHO recommended additional clinical research to ensure the safety of SLD-PQ in G6PDd individuals in different eco-epidemiological settings [20, 28]. Furthermore, due to pragmatic dosing techniques, some patients may receive a dose greater than the recommended dose of 0.25 mg/kg, which may improve gametocyte clearance but increase the risk of haemolysis [29].

Unfortunately, most studies were clinical trials that lacked population-based data and merely provided quantitative data assessing the effect of the SLD-PQ on haemoglobin (Hb) levels. In addition, previous safety studies were often based on carefully chosen study participants and small groups with relatively high pretreatment Hb levels; therefore, they offered limited information at the population level. The risk of anaemia is likely to increase in individuals with lower pretreatment Hb concentrations [30, 31]. Although PQ is linked to a brief reduction in Hb levels in G6PDd patients, baseline Hb levels continue to be the leading cause of anaemia in such patients [9]. Additionally, severe haemolytic events might be rare and unlikely to be observed in small safety studies [26]. Therefore, whether certain groups are still at risk of clinically significant haemolysis when SLD-PQ treatment occurs at the population level needs to be examined. For noncurative treatments that aim to help the community rather than just the dosed individual, this safety profile is particularly crucial [9]. To address this issue, we evaluated the safety of adding a single fixed low dose of PQ (15 mg tablet or 0.25 mg/kg for a person weighing 60 kg) to ACT regimens of artemether-lumefantrine (AL) to treat patients with *P. falciparum* malaria in an Ethiopian cohort.

Moreover, changes in the Hb concentration over time were also examined, which is helpful information for clinical practice. Furthermore, previous studies have mainly examined one population group—males [26], children [32], adults [6] or asymptomatic people [33]—while excluding patients with G6PDd [3]. In the present study, all groups were included.

## Methods

### Study area

**Fig. 1:**
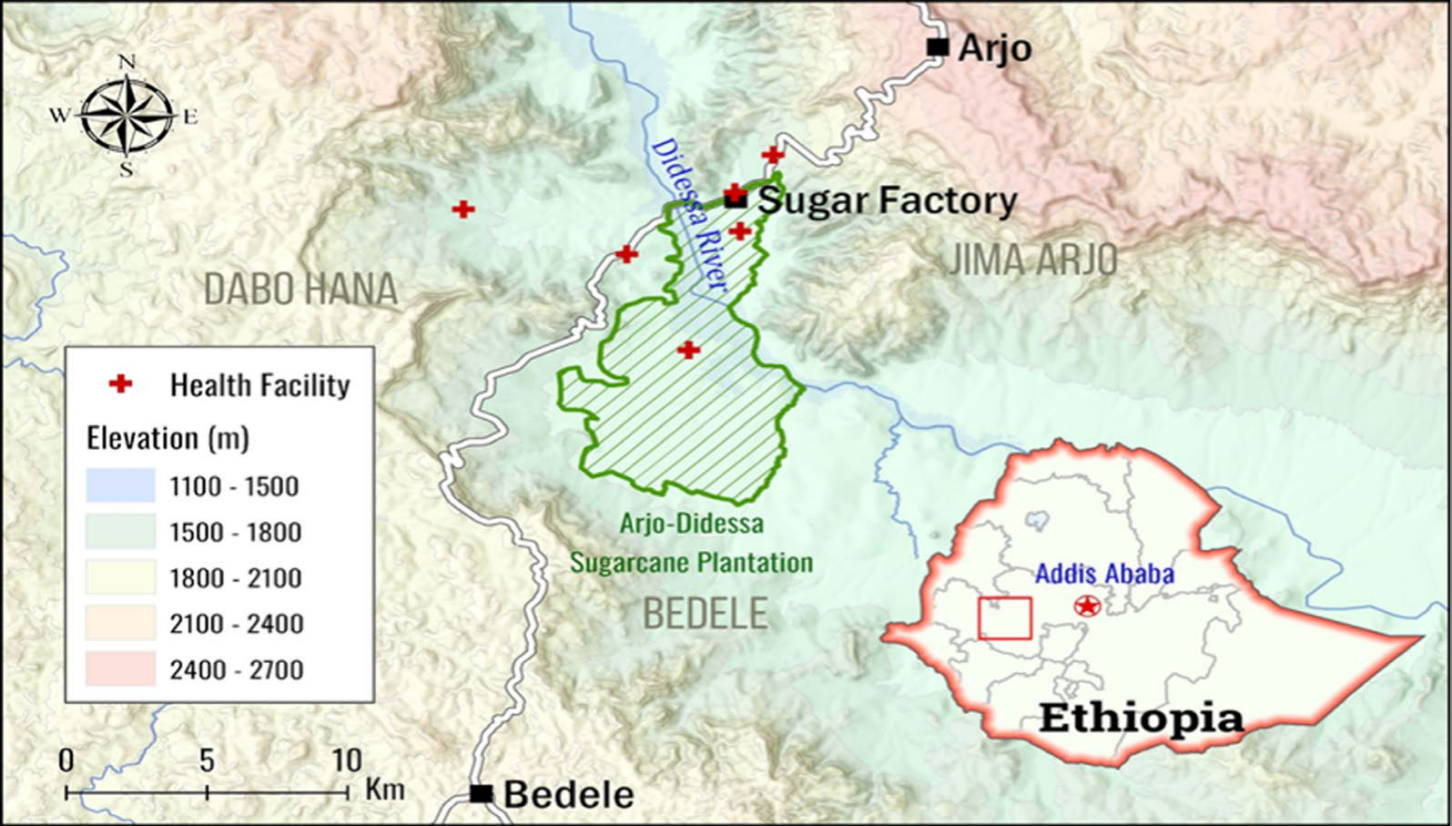
Map of the study sites

This study was conducted at the Arjo-Didessa Sugar Cane Plantation and surrounding areas of southwestern Ethiopia between September 2018 and November 2022. Arjo-Didessa is located 540 km southwest of Addis Ababa, the nation's capital. The latitude of the study area is 8°41′35.5″N, and the longitude is 36°25′54.9″E. The mean annual rainfall is 1477 mm, which falls over two rainy seasons: one short season between February and March and the other long rainy season ranging between June and September. These rainy seasons correspond to low and high peak transmission seasons, respectively. The two primary malaria parasite species are *P. falciparum* and *P. vivax* [34, 35]^.^ Studies in this area have reported *P. ovale* infections [36]. The rates of malaria transmission are low, unstable, and seasonal. The vast majority of people living in the area are farmers who raise food crops, including corn, sorghum, nuts, and peppers, and a smaller portion of these people work at the Arjo sugar factory and sugarcane plantations. The residents also maintained livestock to help with their living. The study was carried out in health facilities located in three districts: Dabo Hana (Kerka Health Post and Sefera Tabiya Health Center), Jimma Arjo (Arjo-Didessa Sugar Factory Clinic, Abote Didessa Health Center), and Bedele (Command 2 and Command 5 Health Posts). The selection of health facilities was based partly on how close they were to the follow-up study. Fig. 1 (Map of the study sites).

### Study design

This was a prospective cohort study on the impact of a single low dose of primaquine on the prevention and control of *P. falciparum* malaria. As part of this study, patients treated for uncomplicated *P. falciparum* malaria with either ACT + SLD-PQ at 0.25 mg/kg or ACT alone were enrolled and assessed for the safety of the addition of SLDPQ. Since the study started in September 2018 and SLD-PQ was not fully adopted nationwide, regions that started utilizing SLD-PQ were targeted for the study. As Study participants from other health facilities that did not enrollSLD-PQ use served as a natural control group. By comparing patients receiving ACT alone with those receiving ACT + SLDPQ, this method aids in evaluating the safety of including SLDPQ in the treatment plan. Then, clinical and laboratory evaluations were conducted on days (D) 0, 7, 14, 21, and 28 or on any day of recurrent illness [37]. Patients who satisfied the study's eligibility criteria, such as uncomplicated *P. falciparum* mono-infection verified microscopically, provided consent to adhere to the study protocol and were included in the study.

The study excluded pregnant women, lactating mothers, and children under one year old due to contraindications to PQ in these populations [38]. Any history of severe malaria or warning signs, a recent history of blood transfusion, febrile conditions unrelated to malaria, or a known chronic or serious underlying illness were among the exclusion criteria.

### Sample size calculation

This study took the primary objective—the mean Hb reduction—into consideration when calculating the proper sample size. The Hb reduction calculation in this study was based on a study in Senegal by Tine et al., which showed that adding primaquine (0·25 mg/kg) to ACT reduced the mean Hb concentration from 13.4 to 11.9 g/dl (1.47) [6]. With a 10% loss to follow-up and a clinically meaningful noninferiority margin of 0.3 g/dl, the intended sample size for the effect of SLD-PQ on Hb was 131 participants per group. This made it possible to determine if the study group and the reference group were not significantly different by 80% at the two-tailed 5% significance level.

### Sampling procedure

Following the detection of *P. falciparum* on D0, artemether + lumefantrine (AL) tablets twice daily for 3 days (Coartem [20 mg of artemether and 120 mg of lumefantrine]; Ipca Laboratories Ltd., India) were given for all positive uncomplicated *P. falciparum* patient malaria cases as per the National Malaria Treatment Guidelines. In addition, SLD-PQ + ACT was also administered on D0 in selected health clinics and posts in the study area. Following guidelines from the FMoH, Primaquine phosphate dose: of 0.25 mg base per kg was prescribed according to an age-based, non-weight-based treatment schedule. The fixed dose for adults was 15 mg, which was taken as two tablets containing 7.5 mg each. The drug was divided in accordance with the guidelines for children [39]. Researchers directly observed participants taking the primaquine dose at the time of enrollment in the study. Adherence to the ACT regimen was assessed through self-reported adherence during the second and seventh days of the follow-up.

### Laboratory procedure

Blood samples for malaria diagnosis using microscopy and dried blood spots for PCR analysis were taken prior to treatment. In addition, capillary blood samples (300 microns) were collected for Hb concentration measurement and determination of G6PD enzyme activity. G6PD was measured once on day 0, and the patient was followed up via scheduled appointments on D7, D14, D21, and D28 for Hb status and microscopy examination. Clinical data, including patient sociodemographic information, malaria symptoms, and history of treatment with anti-malarial drugs, were collected during enrollment.

### Microscopy of malaria parasites

According to the WHO malaria microscopy procedure [40], thick and thin smears were taken at patient enrollment and during follow-up, and they were quickly stained with 10% Giemsa. Two different microscopists examined each microscope slide. When there was disagreement about the presence of parasitaemia or whether there was a difference in parasite density of more than 25%, a third independent reading was performed [41]. Accordingly, microscopic examination was carried out on D 0, 7, 14, 21, and 28 using Giemsa-stained blood films at a magnification of 1000x.

### G6PD enzyme and Hb measurements

Blood samples from each study participant were tested for G6PD enzyme and Hb levels in accordance with the manufacturer’s instructions. Hb and G6PD levels were measured using CareSTART™ POCT S1 (Access Bio, Inc., New Jersey, USA). As recommended by the manufacturer, Hb and G6PD activity were checked daily prior to sample collection. Every sample was run twice. International units per decilitre, or IU/dl, are used to express G6PD enzyme activity, which was measured using a G6PD strip. The enzyme activity was normalized using Hb values taken concurrently with the G6PD enzyme test and expressed as IU/g Hb. Quantitative POCTs were performed using “biosensors,” portable electronic devices where disposable test strips were inserted. Within five minutes after blood was added, the device's screen displayed the results as a quantitative readout. An unused test strip was inserted into the biosensor, and an aliquot from each blood sample (5 µL) was then placed on the exposed end of the test strip until the device displayed complete automated sample intake. The running times for the G6PD and Hb tests were 4 min and 10 s, respectively. Using a previously described methodology [18], the adjusted male median (AMM) G6PD activity (100% G6PD activity) for males was used to determine the cutoff values for G6PD deficiency. After excluding males who were severely G6PD-deficient (i.e., had less than 10% of the median G6PD enzyme activity), as advised by an expert panel, the percentage of G6PD activity was determined based on their relative proportion to the adjusted median value of G6PD enzyme activity among the male population [42]. For male study participants, class I was G6PD deficient with less than 30% AMM activity, while class II was normal with more than 30% AMM activity. Individuals with G6PD activity < 30%, 30–80%, or > 80% of the AMM activity in females are regarded as G6PD deficient, intermediate, or normal, respectively [43–46].

### Clinical and safety assessments

Safety assessments in the present study were performed via haematological analysis and recording of all adverse events [47]. All patients were asked without probing about their health after taking their last dose of anti-malarial medication, and the responses were categorized as none, mild, moderate, severe, or life-threatening. The following adverse events (AEs) were recorded: abdominal pain, nausea, vomiting, haemolysis(if dark urine was encountered using a clean container), itching, rash, and any drug-related severe adverse events (SAEs). AEs were graded using the Primaquine Roll Out Monitoring Pharmacovigilance Tool (PROMPT), a tool that is helpful for assessing the safety of AEs and SAEs [25]. Mild, moderate, severe, and life-threatening AEs were further divided into four categories: mild, easily tolerable; no or little interference with daily activities; moderate, low level of inconvenience; more than little interference with daily activities; severe, interrupted regular daily activities, typically incapacitating; and life-threatening, life-threatening consequences, indicating the need for immediate intervention or death events.

### Outcomes

The main goal of the study was to determine how adding SLD-PQ to ACT affects patients’ mean change in Hb levels [48]. In particular, we examined the changes in Hb from Day 0 of baseline to Day 7 after the start of treatment. The mean absolute fall in Hb by Day 7, the mean fractional decline in Hb from Day 0 to Day 7, and the degree of Hb recovery by Day 28 to baseline values were all evaluated in this analysis [22, 49]. Because Day 7 could be the longest day for PQ-induced haemolysis and because earlier observations showed that Hb levels peaked by this day, Day 7 was chosen for assessment [19]. Either an absolute drop of more than 3 g/dL or a fractional reduction of more than 25% was considered a clinically significant Hb decrease [49].

The secondary outcomes of this study were the occurrence of adverse events (AEs), including blood transfusions, AEs that occurred within 7 and 28 days after the administration of primaquine, and other AEs related to the administration of PQ, such as gastrointestinal reactions within 7 days and risk of haemolysis.

### Data management and analysis

The data were entered into a Microsoft Excel data sheet and analyzed using Stata (v17, StataCorp LLC, Texas) and IBM SPSS Statistics 27 software. The paired t-test was employed to assess the significance of mean Hb within groups, while the unpaired t-test was used to evaluate their significance between groups. Absolute and relative changes in Hb concentration from the baseline value were reported during the follow-up. A linear mixed-effects model was used to assess the impact of treatment on Hb drop. This model yielded the treatment difference at each time point and a 95% confidence interval. Primaquine’s effect in G6PD normal and G6PD deficient patients was estimated in an intended subgroup analysis using a model fitted with baseline Hb as a covariate, patient as a random effect, and G6PD status, the interaction between time and G6PD status, and the treatment group and G6PD status at each time point as fixed effects. The significance of all *P* values was assessed using a two-tailed approach, with a threshold set at *P* < 0.05 for statistical significance. Multiple linear regression model was employed,adjusted for age, sex, and baseline Hb concentration to assess whether there was a significant difference in the mean Hb change between G6PD-deficient patients and normal patients following therapy. The reported complaints and adverse events (AEs) in the ACT alone and ACT + SLD-PQ treatment arms were also compared using Fisher’s exact test.

## Results

### Overview of the study

**Fig. 2:**
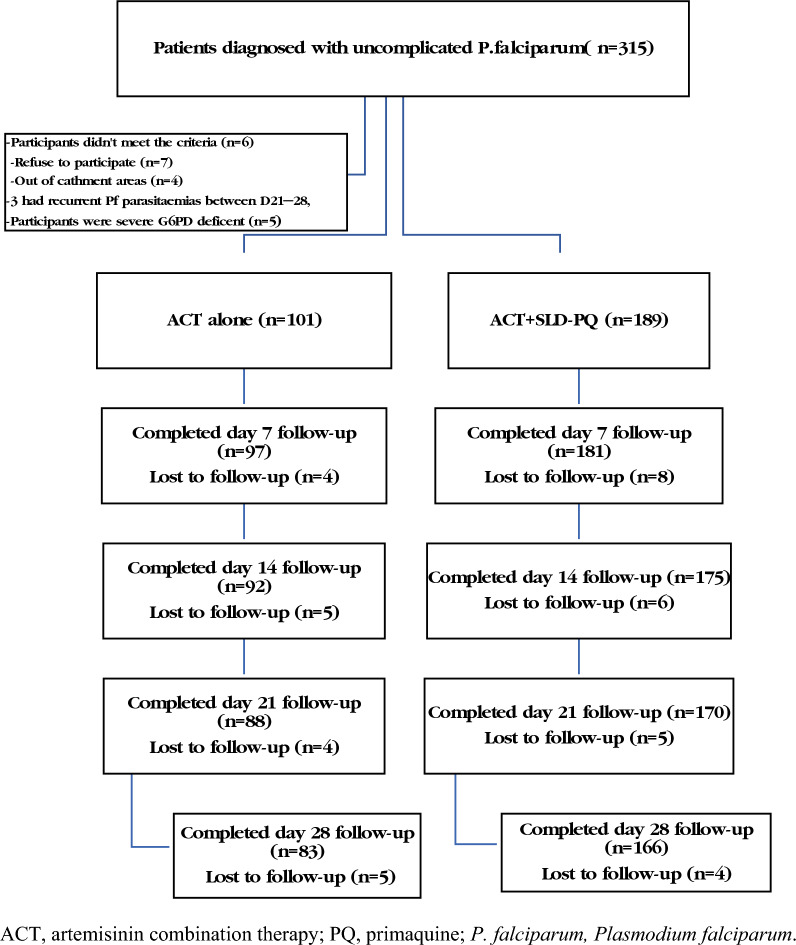
Flow chart of uncomplicated *P. falaciparum* malaria patients enrolled for ACT and SLD PQ + ACT in Arjo Didessa, Southwest Region, Ethiopia. ACT: artemisinin combination therapy, PQ: primaquine, *P. falciparum*: *Plasmodium falciparum*

A total of 315 febrile patients with confirmed falciparum malaria were followed for two seasons during high malaria transmission. The study excluded 66 individuals who were not within the catchment area, did not match the inclusion criteria, had severe G6PDd, or declined to participate (Fig. 2). The remaining 249 (79%) patients were eligible and completed the 28 day follow-up. The initial characteristics of the two treatment groups were comparable (Table 1). Patient sociodemographic characteristics included the total number of patients who received ACT alone (n = 83) or ACT + SLD-PQ (n = 166) for uncomplicated falciparum malaria. Adults (aged > 15 years) accounted for the majority of the study population, with a total of 197 (79.12%) individuals and a sex ratio of males to females of 1.8 (Table 1).

**Table 1:** Baseline sociodemographic, clinical,parasitological profiles and prognostic profiles of the study participants (n = 249) by treatment group in Arjo-Didessa, southern Ethiopia; 2019–2022

Characteristics	Treatment arms		*P *value
	ACT alone 83 (33.33%)	ACT + SLD-PQ 166 (66.67%)	
Gender (% male no. of males/total no. of individuals)	65.06(54/83)	64.46(107/166)	0.9257
Age(years), median (IQR)	20(13)	20 (15)	0.9276
Age groups ≤ 5 years, no. (%) 6–15 years,no.(%) 16–30 years,no.(%) ≥ 31 years,no.(%)	2(2.6) 10(12.8) 49(62.8) 17(21.8)	4(2.5) 32(20.4) 91(58.0) 30(19.1)	0.560
Axillary temperature (^o^C), mean (SD)	38.13(0.54)	37.98(.63)	0.0759
Fever (≥ 37.5 °C) at present, no. (%)	63(75.90)	119(71.69)	
Asexual parasite density/microlitre, mean (SD)	13815.67(19655.76)	17430.92(30420.42)	0.3259
G6PD activity**, no. (%) -Normal -Deficient	77(92.77) 6(7.23)	155(93.37) 11(6.63)	0.859
Hb concentration at enrollment (g/dl), mean (SD)	12.80(1.18)	12.75(0.89)	0.736
G6PDd Hb(g/dl), mean (SD) G6PDn Hb(g/dl), mean (SD)	12.70(0.88) 12.80(1.21)	12.7(0.53) 12.76(.91)	1.0000 0.730

Number [Note: means and standard deviation are presented for temperature and haemoglobin; age is presented as median and interquartile range]

IQR: interquartile range, °C: degree Celsius

^**^ as determined by a POCT analyzer indicating G6PD enzyme activity (IU/g Hb)]

### Phenotypic G6PD status

**Fig. 3:**
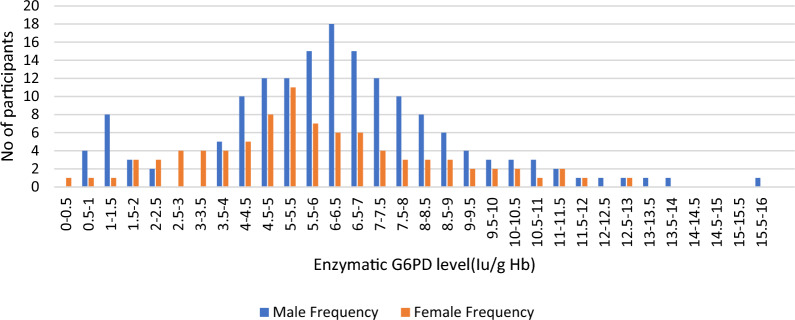
Distribution of G6PD enzyme levels by sex at Arjo-Didessa, southern Ethiopia; 2019–2022

At the time of enrollment, phenotypic point-of-care testing was utilized to screen patients for G6PD deficiency. The adjusted median G6PD enzyme activity was determined for the male participants by excluding 5 patients whose enzyme activity was less than 10% (less than 0.627 U/g Hb) of the median value found for all male participants. The median G6PD activity was 6.27 U/g Hb for all study participants (the range was 0.4–15.22 U/g Hb). The AMM (range) enzyme activity was 6.32 IU/gHb (0.67–15.219), which was 100%. The distribution of enzymatic activity by sex is illustrated in Fig. 3. There was a distinct bimodal distribution in males and a unimodal distribution in females. When the participants’ enzyme activity was between 0.4 and 1.896 U/g Hb (less than 30% of the adjusted male median), they were classified as deficient. Phenotypic G6PD data were recorded for 249 patients; 17 (6.83%) had G6PDd—14 males and 3 females. In the ACT alone arm, there were 6 (2.41%) patients with phenotypic G6PDd, whereas there were 11 (4.42%) patients in the ACT + SLD-PQ arm (Table 1).

### Haemoglobin profiling

Data from 415 Hb measurements in 83 patients treated with ACT alone and 830 Hb measurements in 166 patients treated with ACT + SLD-PQ were used for the assessment of the changes in the mean Hb level between baseline and days 7, 14, 21, and 28. During enrollment, the mean (SD) Hb level was 12.80 (1.18) g/dL for patients receiving ACT alone and 12.75 (0.89) g/dL for those receiving ACT + SLD-PQ (Table 1). There was no significant difference in the baseline Hb levels between the two treatment arms (*p* = 0.7357). However, males had greater mean (SD) Hb concentrations at baseline (12.78 (1.03) [95% CI 12.62, 12.94]) than females did (12.75 (0.93) [95% CI 12.55–12.94]). There were no significant differences in the mean Hb concentration between males and females in the two treatment arms (*p* = 0.8281). However, as illustrated in Table 3, in both groups receiving ACT + SLD-PQ, the mean Hb concentrations, shown as the mean change, decreased in the first week following treatment and reverted to baseline values during follow-up. Similarly, paired analysis of Hb concentrations relative to baseline demonstrated a reduction in the first week after receiving 0.25 mg/kg PQ + ACT or ACT alone. These reductions were significant on day 7 following SLD-PQ (0.25 mg/kg PQ) (*P* < 0.01) or ACT alone (*P* < 0.01; Table 2). However, there was no significant difference between the treatment groups, ACT alone and ACT + SLD-PQ (*P* = 0.157; Table 3).

**Table 2:** Mean change and mean haemoglobin concentration at baseline and day 7 in the treatment group in Arjo-Didessa, southern Ethiopia; 2019–2022

Treatment groups	N	Mean (baseline)	Mean (on day 7)	Mean diff.	t-value	*P* value
ACT alone	83	12.80	12.49	0.30	3.6	0.0005*
ACT + SLD-PQ	166	12.75	12.30	0.45	13.75	0.0000*

[Note: n, number of observations; mean baseline, mean of baseline haemoglobin concentration in g/dl; mean on day 7, mean of day 7 follow-up time haemoglobin concentration in g/dl; Diff; mean difference (95% CI) estimated using a paired Student t test comparing the mean concentration of haemoglobin on day 7 in each group]

ACT: artemisinin combination therapy, PQ: primaquine

**Table 3:** The mean Haemoglobin concentration in G6PD deficient and normal patients at each time point, the mean and the change from baseline, and the adjusted effect of a single low dose primaquine on the change from baseline estimated from the linear mixed effects model

	G6PD	Mean Hb concentration in g/dL (SD)		Change from baseline mean (SD)		Adjusted difference in change from baseline (95% CI)	
Days	status	ACT alone	ACT + SLD-PQ	ACT alone	ACT + SLD-PQ	(ACT + SLD-PQ) − (ACT alone)	*P*-Value**
0	Normal	12.80 (1.21)	12.76 (0.91)	*	*	*	*
	Deficient	12.70 (0.88)	12.70 (0.53)	*	*	*	*
7	Normal	12.51 (1.18)	12.31 (0.97)	− 0.29 (0.79)	− 0.44 (0.43)	0.15 (− 00, 0.31)	0.056
	Deficient	12.22 (0.86)	12.10 (0.56)	− 0.48 (0.21)	− 0.60 (0.34)	0.12 (− 0.22, 0.45)	0.465
14	Normal	12.77 (1.03)	12.67 (0.79)	− 0.04 (0.81)	− 0.09 (0.68)	0.05 (− 0.15, 0.25)	0.599
	Deficient	12.47 (0.89)	12.44 (0.72)	− 0.23 (0.40)	− 0.26 (0.66)	0.03 (− 0.60, 0.67)	0.9203
21	Normal	12.86 (0.97)	12.72 (0.83)	0.06 (0.93)	− 0.03 (0.61)	0.09 (− 0.11, 0.29)	0.383
	Deficient	12.57 (0.94)	12.53 (0.72)	− 0.13 (0.52)	− 0.17 (0.62)	0.04 (− 0.60, 0.68)	0.8970
28	Normal	12.93 (0.94)	12.76 (0.86)	0.13 (0.97)	0.01 (0.54)	0.12 (− 0.07, 0.32)	0.2126
	Deficient	12.68 (0.93)	12.59 (0.74)	− 0.02 (0.66)	− 0.11 (0.69)	0.09 (− 0.64, 0.83)	0.7919

^*^Baseline Hb values only

^**^The *P*-value is from a test of interaction between G6PD status and treatment

After posttreatment (D7), Hb levels decreased on average by 0.45 g/dl (95% CI = 0.39 to 0.52) in patients receiving ACT + SLD-PQ compared to 0.30 g/dl (95% CI = 0.14 to 0.47) in patients receiving ACT alone (*P* = 0.157; Table 3). Patients with and without SLD-PQ experienced overall (D0–14) Hb losses of 0.10 g/dl (95% CI = − 0.00 to 0.20) and 0.05 g/dl (95% CI = − 0.123 to 0.22), respectively (*P* = 0.412; Table 3). At the overall follow-up (D0-28), the mean Hb levels were marginally lower in patients treated with ACT + SLD-PQ than in those not treated with ACT + SLD-PQ. Patients in the ACT alone and ACT + SLD-PQ arms recovered their Hb to or above the baseline values by day 28.

### Mean Hb (SD) changes following treatment

The overall pattern showed an initial rapid drop in Hb levels followed by a lengthy recovery period; however, the difference between the two arms was not statistically significant (*p* = 0.157). On day 7, the mean Hb reduction was comparable between the groups.

### Hb reduction by G6PD phenotype and treatment arm

To address concerns about haemolysis associated with SLD-PQ use in G6PDd individuals, Hb concentrations were assessed at enrollment and throughout follow-up. When all the G6PDd patients were phenotypically combined, compared with those in G6PDn patients, the mean Hb reduction was − 0.24 g/dl (*P* = 0.359). Overall, the baseline mean (± SD) Hb concentrations were similar between G6PDd patients and G6PDn patients [12.77 (1.01) g/dL vs. 12.70 (0.64) g/dL] and during each follow-up period [D7 12.14 (0.66) g/dL vs. 12.38 (1.05) g/dL; D14 12.45 (0.75) g/dL vs. 12.70 (0.87) g/dL; D21 12.54 (0.78) g/dl vs. 12.77 (0.88) g/dL; D28 12.62 (0.79) g/dl].

### Reduction in Hb levels from Day 0 to Day 7 based on phenotypic G6PD status and treatment arm

The mean Hb concentrations, expressed as an absolute or relative change, decreased in the first week following treatment in all groups receiving ACT + SLD-PQ and recovered to baseline levels at follow-up were presented (Tables 4 and 5). Collectively, patients identified as phenotypic G6PDd did not exhibit any statistically significant difference in Hb reduction compared to G6PDn patients. A further breakdown of the absolute mean Hb reduction by phenotypic G6PD status is presented in Table 4. For patients treated with ACT alone, the absolute mean Hb reduction among G6PDn and G6PDd individuals was [0.26 g/dL (95% CI: − 0.73, 0.21), *P* = 0.279]. In the ACT + SLD-PQ arm, corresponding results were 0.34 g/dL (95% CI: − 0.70, 0.01, *P* = 0.058) and 0.16 g/dL (95% CI: − 0.31, − 0.00, *P* = 0.05), respectively. No statistically significant differences was observed in absolute mean Hb reduction compared to G6PDn patients treated with ACT alone.

**Table 4:** Mean absolute Haemoglobin reduction between days 0,7,14,21 and 28 by phenotypic G6PD status & treatment arm

Treatment group				
	G6PD normal		G6PD deficient	
Mean absolute Hb reduction (g/dL) (95% CI)	ACT alone (n = 77)	ACT + SLD-PQ (n = 155)	ACT alone (n = 6)	ACT + SLD-PQ(n = 11)
Day 7, Hb change, g/dL, mean (SD) Compared to G6PD normal ACT alone, g/dL, mean difference (95% CI) *P value	− 0.29 (0.79)	− 0.44 (0.43) − 0.16 (− 0.31, − 0.00) 0.047	− 0.48 (0.21) − 0.26 (− 0.73, 0.21) 0.279	− 0.60 (0.34) − 0.34 (− 0.70, 0.01) 0.058
Day 14, Hb change, g/dL, mean (SD) Compared to G6PD normal ACT alone, g/dL, mean difference (95% CI) *P value	− 0.04 (0.81)	− 0.09 (0.68) − 0.05 (− 0.25, 0.14) 0.588	− 0.23 (0.40) − 0.24 (− 0.84, 0.37) 0.437	− 0.26 (0.66) − 0.25 (− 0.71, 0.21) 0.284
Day 21, Hb change, g/dL, mean (SD) Compared to G6PD normal ACT alone, g/dL, mean difference (95% CI) *P value	0.06 (0.93)	− 0.03 (0.61) − 0.09 (− 0.29, 0.11) 0.366	− 0.13 (0.52) − 0.26 (− 0.86, 0.34) 0.398	− 0.17 (0.62) − 0.27 (− 0.72 to 0.19) 0.255
Day 28, Hb change, g/dL, mean (SD) Compared to G6PD normal, g/dL, mean difference (95% CI) *P value	0.13 (0.97)	0.01 (0.54) − 0.13 (− 0.32, 0.07) 0.199	− 0.02 (0.66) − 0.23 (− 0.83, 0.36) 0.436	− 0.11 (0.69) − 0.28 (− 0.73, 0.16) 0.212

^*^The *P*-value is from a test of interaction between G6PD status and treatment from the Linear mixed effect model

**Table 5:** Mean relative Haemoglobin reduction between days 0,7,14,21 and 28 by phenotypic G6PD status & treatment arm

Treatment Group				
	G6PD Normal		G6PD Deficient	
Mean fractional Hb reduction (%) (95% CI)	ACT alone (n = 77)	ACT + SLD-PQ (n = 155)	ACT alone (n = 6)	ACT + SLD-PQ(n = 11)
Day 7, Hb change, %, mean (SD) Compared to G6PD normal, g/dL, mean difference (95% CI) *P* value	− 2.07 (6.28)	− 3.48 (3.34) − 1.42 (− 2.62, − 0.21) 0.021	− 3.80 (1.63) − 2.25 (− 5.94,1.44) 0.231	− 4.72 (2.59) − 2.91 (− 5.70, − 0.11) 0.041
Day 14, Hb change, %, mean (SD) Compared to G6PD normal, g/dL, mean difference (95% CI) *P* value	0.09 (7.51)	− 0.48 (5.51) − 0.58 (− 2.27, 1.11) 0.502	− 1.81 (3.07) − 2.24 (− 7.42, 2.94) 0.395	− 2.02 (5.37) − 2.29 (− 6.21, 1.63) 0.252
Day 21, Hb change, %, mean (SD) Compared to G6PD normal, g/dL, mean difference (95% CI) *P* value	0.89 (8.33)	− 0.10 (4.93) − 1.00 (− 2.69, 0.69) 0.243	− 1.02 (3.99) − 2.50 (− 7.67, 2.67) 0.342	− 1.32 (5.05) − 2.52 (− 6.44, 1.39) 0.206
Day 28, Hb change, %, mean (SD) Compared to G6PD normal, g/dL, mean difference (95% CI) *P* value	1.54 (8.68)	0.16 (4.31) − 1.39 (− 3.04, 0.26) 0.098	− 0.04 (5.15) − 2.33 (− 7.38, 2.72) 0.365	− 0.80 (5.58) − 2.73 (− 6.55, 1.10) 0.162

G6PDdd: glucose-6-phosphate dehydrogenase deficiency, G6PDn: normal glucose-6-phosphate dehydrogenase

#### Hb recovery

By Day 28, 38 out of 83 patients (45.8%) in the ACT alone arm and 71 out of 166 patients (42.8%) in the ACT + SLD-PQ arm had recovered their Hb levels. No statistically significant difference was found in the proportion of patients with Hb recovery by day 28 compared to G6PDn patients treated with ACT alone (*P* = 0. 0.212). Again, there was no significant difference in mean Hb recovery between the G6PDd ACT alone and the G6PDd ACT + SLD-PQ arms at day 28, even though there was no documented Hb concentration recovery in either treatment arm (*P* = 0.792).

#### Relative Hb reduction

Table 5 presents the percentage relative mean Hb reductions based on phenotypic G6PD status. For patients treated with ACT alone, the relative mean Hb reduction in G6PDd individuals was 2.25% (95% CI − 5.94, 1.44, *p* = 0.231). In contrast, significant differences were observed in the relative mean Hb reduction between G6PDd and G6PDn patients treated with ACT + SLD-PQ compared to G6PDn patients treated with ACT alone (2.91% [95% CI − 5.70, − 0.11], *p* = 0.041 and 1.42% [95% CI − 2.62, − 0.21], *p* = 0.021, respectively). After adjusting for baseline parasitaemia, Hb levels, age, and sex, Table 5 shows a significant reduction in relative Hb for G6PDd and G6PDn patients treated with ACT + SLD-PQ.To potentially better capture haemolysis, maximum decreases in Hb concentration(the largest reduction in Hb compared to baseline at any time point during follow-up were assessed on day 7). after adjustment for baseline Hb concentration. Participants with G6PDd exhibited larger maximum drops in Hb concentration compared to G6PDn participants, both in absolute (mean difference, − 0.60 g/dL; 95% CI − 0.70, − 0.01, *P* = 0.058) and relative (mean difference, − 4.72 g/dL; 95% CI − 5.70, − 0.11, *P* = 0.041) terms. Moreover, none of the patients experienced acute haemolysis during follow-up, which is defined as a Hb drop of absolute (> 3 g/dl) or fractional(> 25%) Hb concentration reductions were seen in any of the treatment arms.

### Mean Hb concentration reduction by G6PD phenotype

A paired t-test model showed a negative association between D7-D0, D14-D0, D21-D0, and D28-D0 decreases in the G6PDd and G6PDn treatment groups, but G6PDn, in comparison to G6PDd, was linked to a positive change in D28-D0 Hb, with a mean increase of 0.05 g/dl (95% CI − 0.14, *P* = 0.316). The decrease in the mean Hb concentration after D 7 was greater in the G6PDd group than in the G6PDn group, but the difference was not significant (*P* = 0.359): − 0.56 g/dl vs. − 0.39 g/dL, respectively; ΔHb concentration = − 0.24 (− 0.75, 0.27) g/dL. Table 3 shows the reduction in the mean Hb concentration according to the G6PD phenotype. On day 14, the mean decreases in Hb in the G6PDn and G6PDd groups were nearly equal to their values from the previous day, which were − 0.07 g/dl and − 0.25 g/dl, respectively. On day 21, however, there was no mean Hb decrease in G6PDn individuals, but there was a mean Hb decrease of − 0.16 g/dl in G6PDd individuals. On day 28, the mean Hb reduction in G6PDd patients was − 0.102 g/dl; however, the mean increase in Hb in G6PDn patients was 0.05 g/dl. This difference was not significant (*P* = 0.337).

**Fig. 4:**
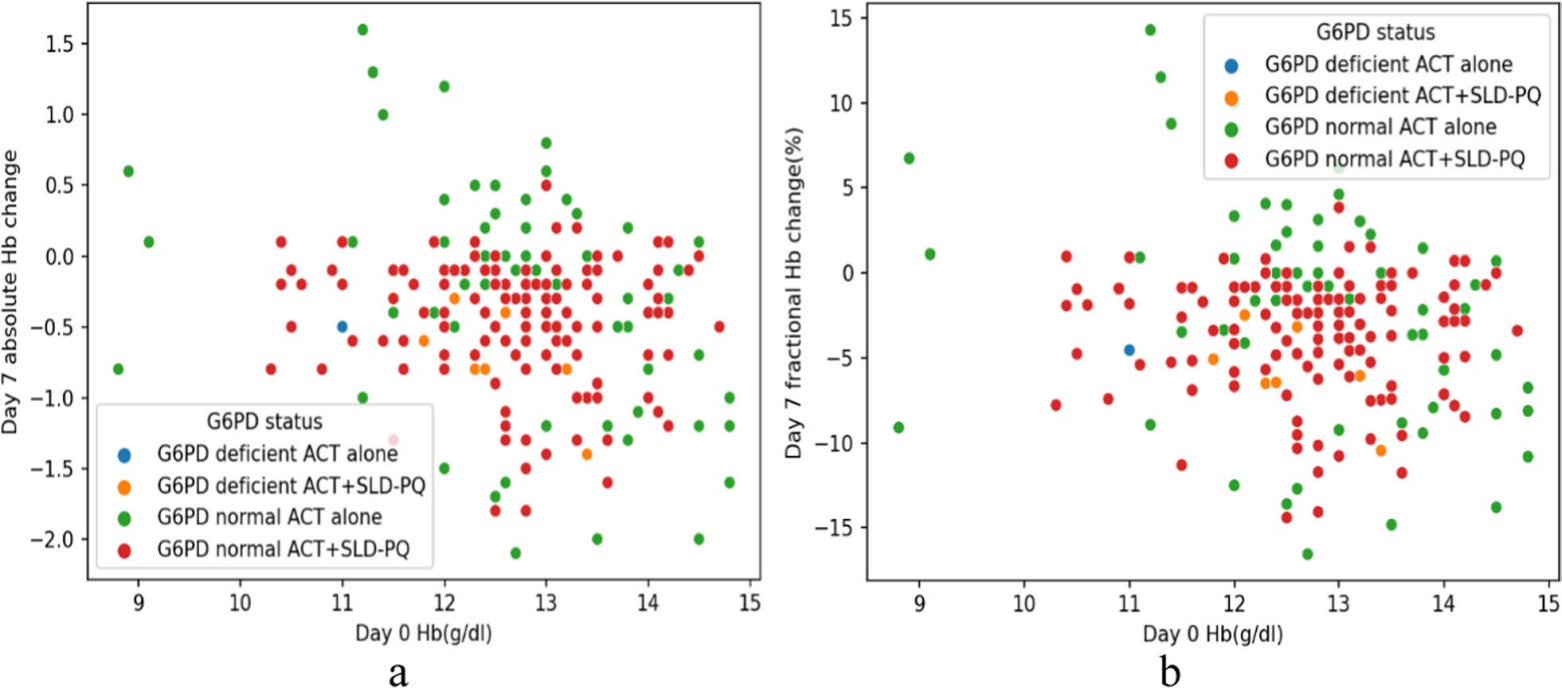
Change in absolute Hb concentration by day 7(Hb day 7-day 0) (**a**) and change in fractional Hb concentration by day 7[100*(absolute Hb /baseline Hb)] (**b**) plotted against the concentration of Hb at baseline, for each group

In comparison to those with G6PDn ACT alone, both the ACT alone and the ACT + SLD-PQ G6PDd cohorts experienced a lower mean Hb concentration, with mean changes ranging from 0.45 g/dl [95% CI − 0.486 to 0.079] to 0.54 g/dl [95% CI − 1.067 to 0.238] (Table 5 and Fig. 4). In addition, on day 7 following ACT + SLD-PQ treatment, the Hb concentration in these G6PDd participants ranged from 12.70 to 12.10 g/dL, and that in the ACT-alone G6PDd participants ranged from 12.70 to 12.22. The distribution of patients between treatment arms was unaffected by sex or phenotypic G6PD phenotype. Although the mean changes in Hb in these groups were greater than those in the G6PDn ACT treatment group, a significant difference in Hb levels was not detected on Day 7 posttreatment for G6PDd ACT + SLD-PQ vs. G6PDn ACT alone (*P* = 0.109, *P* = 0.304; Table 5).

**Fig. 5:**
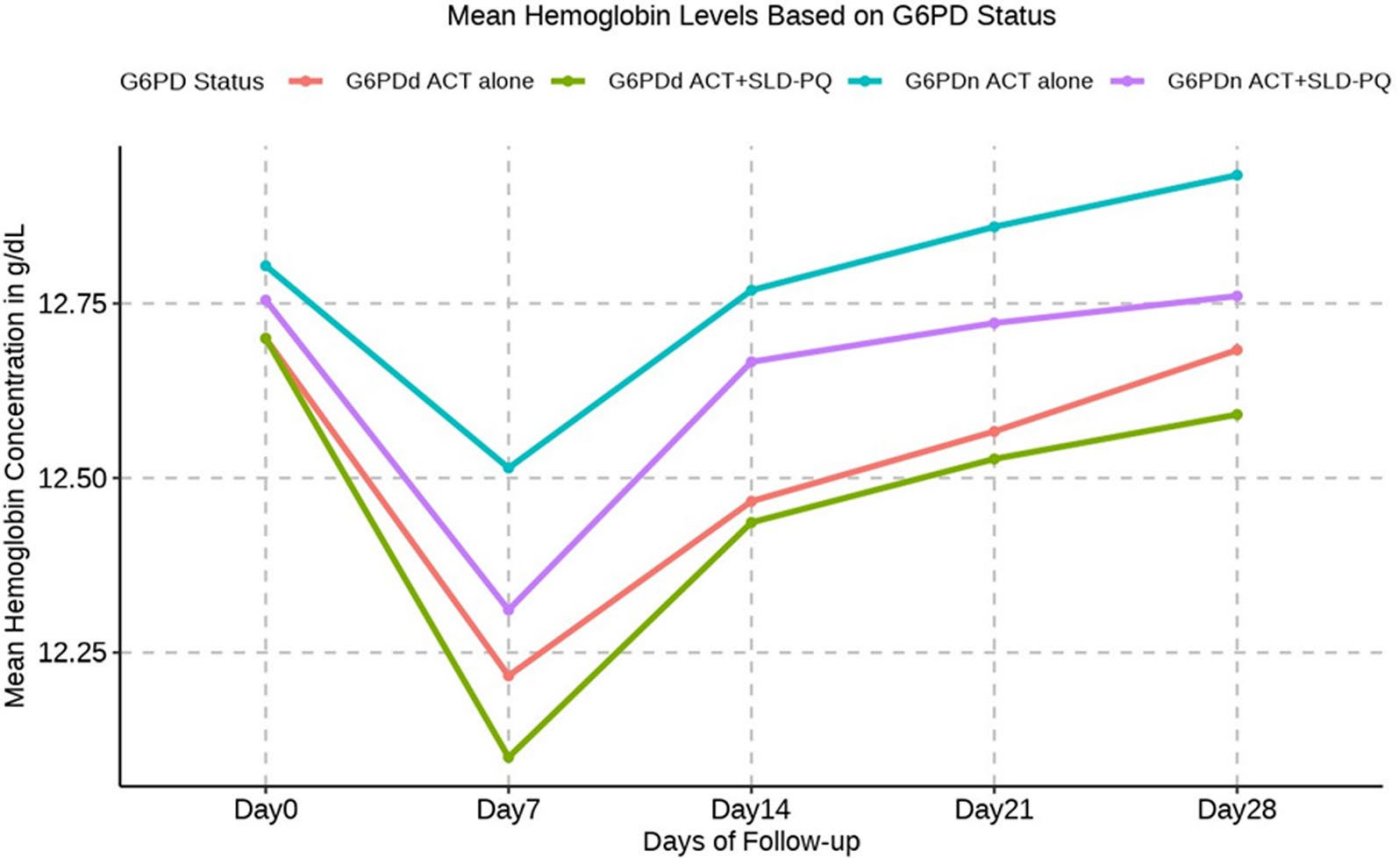
Mean haemoglobin concentrations in G6PDd and G6PDn study participants who received ACT + SLD-PQ or ACT alone

However, the absolute mean reduction in Hb levels on day 7 was not significantly lower in either G6PDd (− 0.54 g/dl 95% CI − 1.19, 0.12; *P* = 0.109) or G6PDn (− 0.20 g/dl 95% CI − 0.48, 0.08; *P* = 0.154) ACT + SLD-PQ individuals (Table 5). There were consistently lower Hb concentrations in G6PDd participants treated with ACT + SLD-PQ than in G6PDn participants, although these differences were not clinically significant Fig. 5.

### Adverse events in the treatment groups

In the study, abdominal pain, appetite loss, fatigue, and nausea were the major AEs, followed by skin rash, cough, headache, diarrhoea, and vomiting. Out of the 249 study participants who completed the follow-up, 94 AEs were recorded—51 (30.7%) from the ACT + SLD-PQ cohort and 43 (51.8%) from the ACT alone group (Table 6). All of the reported AEs were rated as mild. The difference in the incidence of adverse events (AEs) between the treatment groups was not significant (Table 6). There were no SAEs in either treatment group. None of the participants required blood transfusions. In addition, during the study follow-up, every adverse event (AE) resolved, and none of the adverse events stopped participating.

**Table 6:** Adverse events (AEs) among study participants by G6PD status and treatment group, Arjo Didessa, Ethiopia

	Treatment arms				
Adverse events	ACT only		ACT + SLD-PQ		*P* value
	G6PDn n (%)	G6PDd n (%)	G6PDn n (%)	G6PDd n (%)	
Head ache	4 (11.43%)	0	1 (2.38%)	1 (11.11%)	0.097
Nausea	6 (17.14%)	1 (12.5%)	4 (9.52%)	1 (11.11%)	0.062
Vomiting	1 (2.86%)	0	1 (2.38%)	1 (11.11%)	0.705
Abdominal pain	5 (17.14%)	2 (25%)	12 (28.57%)	2 (22.22%)	0.460
loss of appetite	5 (14.29%)	2 (25%)	9 (21.43%)	1 (11.11%)	0.321
Fatigue	6 (17.14%)	1 (12.5%)	5 (11.91%)	1 (11.11%)	0.098
Skin rashes	2 (5.71%)	1 (12.5%)	5 (11.91%)	1 (11.11%)	0.626
Cough	4 (11.43)	1 (12.5%)	3 (7.14%)	0	0.084
Diarrhea	1 (2.86%)	0	2 (4.76%)	1 (11.11%)	0.593
Total	35 (100%)	8 (100%)	42 (100%)	9 (100%)	

## Discussion

This observational cohort study's findings provide significant new insights into the safety profile of ACT + SLD-PQ combination therapy for managing uncomplicated falciparum malaria in individuals with G6PDd from Ethiopia. This study is the first of its kind in which patients with confirmed enzymatic G6PDd were included in an effort to more accurately reflect real-world circumstances. Further proof of the haematological effects of this treatment regimen in the PQ cohort is provided by the study using longitudinal panel measurements from the Arjo-Didessa. Interestingly, a week after starting treatment, there was a noticeable drop in the mean Hb concentration, suggesting a temporary haematological effect. However, it was also observed that Hb levels returned to baseline values by the 28th day of treatment, indicating a recuperative trend during treatment. It is remarkable, nonetheless, that the G6PD deficit did not exhibit the same pattern.

Various studies examining the relationship between G6PDd and posttreatment haemolysis induced by oxidant anti-malarial drugs have used Hb concentration (mean decrease) as the primary outcome measure because changes in Hb levels are objectively measurable [20, 24, 26, 27]. According to the findings in this study, there was no statistically significant difference (*P* = 0.157) between the treatment groups that got ACT + SLD-PQ and ACT-alone. Results from an earlier study conducted in Burkina Faso are similar, supporting the idea that there is a consistent pattern in this context [29]. Further investigation is necessary in light of the noted decrease in Hb levels among individuals who were only given ACT. There are various reasons related to the pathophysiology of malaria and its treatment that could be responsible for this reduction. The loss of red blood cells carrying parasites during schizont rupture may be the reason for the decrease in Hb levels in the ACT alone group. Furthermore, haemolysis is caused by systemic inflammation and oxidative stress brought on by the malaria infection, which leads to the loss of both parasitized and non-parasitized red blood cells [50–53].

Similarly, the mean changes in Hb concentration concerning baseline values and the mean variations in Hb concentration concerning the status of G6PD were further analysed. The findings showed that in all groups treated with ACT + SLD-PQ, mean Hb concentrations, whether expressed as absolute values or relative changes, decreased during the first week of therapy and recovered to baseline levels during follow-up. A week after the administration of ACT + SLD-PQ, G6PDd participants showed a decrease in their mean Hb concentration compared to their baseline values. On day 7, there was no significant difference in the mean Hb levels between the patients treated with ACT-alone and those treated with ACT + SLD-PQ (coefficient, − 0.45; 95% CI − 1.01, 0.10; *P* = 0.108). According to a previous study that assessed the safety of this SLD-PQ as a transmission-blocking therapy showed comparable decreases in Hb levels, Hb levels in G6PDd patients temporarily decreased after they were treated with ACT + SLD-PQ [29]. The findings of this study were consistent with those of previous studies indicating low safety concerns associated with SLD-PQ in G6PDd malaria patients [24, 26, 54, 55]. Additionally, SLD-PQ treatment was linked to a temporary drop in Hb levels in G6PDd individuals [54]. Similarly, a recent systematic review [56] reported that the haemolytic effects of SLD-PQ (0.1 and 0.25 mg/kg) were less likely in individuals who received G6PDd than in those who received a previous dose of 0.75 mg/kg. Similarly, a recent systematic review [56] reported that the haemolytic effects of SLD-PQ (0.1 and 0.25 mg/kg) were less likely in individuals who received G6PDd than in those who received a previous dose of 0.75 mg/kg. Overall, these findings imply that reduced G6PD enzyme activity may not lead to a considerable reduction in Hb levels following day 7 after ACT + SLD-PQ therapy when only given at the recommended dose. Contrasting results are reported in a study in Senegal [6], where patients with G6PDd on Day 7 post-treatment had considerably lower Hb levels than G6PDn persons. This variation highlights the variety in haematological responses to antimalarial medication among various populations and settings, implying the possibility of regional or population-specific variables affecting the dynamics of Hb after treatment.

In this study, there were no significant differences seen in the mean absolute reduction in Hb levels by day 7 after treatment initiation based on G6PD status. G6PDd patients treated with ACT alone showed an absolute mean Hb decrease of 0.26 g/dL (95% CI − 0.73, 0.21, *P* = 0.279). On the other hand, the similar results for G6PDn and G6PDd patients in the ACT + SLD-PQ arm were [0.34 g/dL (95% CI − 0.70, 0.01, *P* = 0.058) and 0.16 g/dL (95% CI − 0.31, − 0.00, *P* = 0.05)], respectively. Comparing the absolute mean Hb reduction to G6PDn individuals treated with ACT alone, no statistically significant changes were found. The results of this investigation are consistent with earlier studies that looked at the safety of SLD-PQ with G6PD level [23, 26]. Other research, however, has demonstrated a notable drop in absolute mean Hb levels between the treatment arms although they did not compare the treatment outcomes by G6PD status [25]. This shows that early in the course of treatment, there is a consistent haematological response to antimalarial therapy. Comparing G6PDd patients with normal G6PD activity and those who received ACT alone or in combination with SLD-PQ, similar trends were found [26]. Moreover, the absence of acute haemolysis, which is characterized by a drop in fractional Hb levels of > 25% or a decrease in 3 g/dl or above during the follow-up, as evidenced by the absence of significant drops in Hb levels, indicates the safety of both treatment arms in terms of haemolytic risk.

This study also provides significant inverse data in that most of the available studies did not investigate the relative mean Hb concentration decline in G6PDn patients who were treated with primaquine in particular. These results demonstrate that, when treated with SLD-PQ, individuals with different G6PD statuses had significantly different relative mean Hb levels. There was not a significant distinction in the relative mean Hb reduction between G6PDd and G6PDn individuals receiving ACT alone. In contrast to G6PDn patients treated with ACT alone, both G6PDd and G6PDn patients treated with SLD-PQ and ACT showed a significant relative mean Hb reduction (2.91% [95% CI − 5.70, − 0.11],* P* = 0.041, and 1.42% [95% CI − 2.62, − 0.21], *P* = 0.021, respectively). The precise reasons behind the observed decrease in Hb levels in patients receiving combination therapy remain unclear.

The study also revealed that by Day 28, a comparable proportion of patients in both the ACT alone arm and ACT + SLD-PQ arm had recovered their Hb levels. This lack of statistically significant difference *(p* = 0. 0.212) suggests that the addition of SLD-PQ to the treatment regimen did not significantly affect Hb recovery rates in G6PDn patients. These results align with earlier research on Tanzanian individuals with uncomplicated *P. falciparum* infections. These earlier studies likewise reported a decrease in Hb levels on Day 7 after the start of treatment, followed by a period of recovery [23]. Mean changes in Hb concentration provided a broad comparison between treatment groups; maximal reductions might more accurately represent extreme haemolysis [57]. The baseline Hb concentration was taken into account when comparing the results, and the maximum reductions in Hb concentration (the largest reduction in Hb concentration compared to baseline at any time point during follow-up) were greater in both the ACT alone and ACT + SLD-PQ groups on Day 7 posttreatment, with a relative mean difference of − 0.20 g/dL (95% CI − 0.08, 0.47; *P* = 0.157). The study did not show any significant differences between the two groups in terms of the maximum reductions in Hb concentration, which was predicted [58]. There were no statistically significant differences in the absolute mean maximal reduction in Hb concentration or the relative mean maximal reduction in Hb concentration between G6PDd patients receiving ACT + SLD-PQ and ACT alone (*P* = 0.058 and 0.066, respectively). The results of the current study align with what has been observed in similar research studies before [22]. It is important to keep in mind, nevertheless, that data from other research have suggested that G6PDd patients showed larger Hb falls compared to G6PDn in terms of their mean fractional Hb changes [26, 59]. Overall, these findings imply that reduced G6PD enzyme activity may not lead to a considerable reduction in Hb levels following day 7 after ACT + SLD-PQ therapy when only given at the recommended dose.

The biological plausibility of haemolysis caused by primaquine in patients with G6PDd demonstrates complex relationships between drug metabolism and red blood cell function. The liver metabolizes primaquine, one of the most important anti-malarial medications, producing reactive metabolites that cause oxidative stress in red blood cells [60]. Red blood cells with G6PDd are more susceptible to oxidative damage because their defense systems are compromised, resulting in insufficient enzymatic activity. Due to this increased susceptibility, those with G6PD deficiency are more likely to have haemolysis from primaquine because the delicate balance between antioxidants and oxidants is tilted in favour of cellular damage [61]. This possibility is supported by clinical data, which includes reports of haemolytic responses in G6PDd individuals after primaquine treatment. Nonetheless, the suggestion by the World Health Organization that a single low dose of primaquine be used in the fight against malaria is part of a larger public health strategy that aims to eliminate malaria [62]. However, recent research—including the one that was mentioned—supports the safety of primaquine in those who lack G6PD and offers a detailed analysis of the drug's risk–benefit ratio [27].

One limitation of this study was the small number of G6PD-deficient study participants, and an unequal number of study participants were followed from the two study arms. Furthermore, determining Hb levels on Day 3 would have yielded a more thorough comprehension of the nadir Hb. Moreover, future studies employing sizable population-based cohorts are recommended to strengthen the robustness of the conclusions about the safety of SLD-PQ in G6PD-deficient individuals.

## Conclusion

This study's findings indicate that using SLD-PQ in combination with ACT is safe for uncomplicated falciparum malaria regardless of the patient’s G6PD status in Ethiopian settings. Caution should be taken in extrapolating this finding in other settings with diverse G6DP phenotypes.

## Data Availability

The data that support the findings of this study are available from the corresponding author, [Kassahun Habtamu], upon reasonable request.

## Appendix

See Tables 7, 8.

**Table 7:** Results of multiple linear regression showing the mean Hb concentration and range of change from baseline for patients with and without G6PD deficiency on day 7

Day 7	Coef.	t value	[95% CI]	P value
Sex: base male	0			
Female	− 0.23	− 1.64	− 0.50–0.05	0.102
Age	0.01	1.71	− 0.00–0.02	0.089
G6PD status				
G6PDd ACT alone	− 0.45	− 1.03	− 1.31–0.41	0.304
G6PDn ACT + SLD-PQ	− 0.20	− 1.43	− 0.48–0.08	0.154
G6PDd ACT + SLD-PQ	− 0.54	− 1.61	− 1.19–0.12	0.109

Coef is the mean Hb coefficient of variation estimated using a multiple linear regression model comparing the mean reduction in Hb concentration on day 7 in each group

G6PD-d: glucose-6-phosphate dehydrogenase deficiency, G6PD-n: normal glucose-6-phosphate dehydrogenase

**Table 8:** The mean haemoglobin concentration and range of change from baseline to day 14 for patients with and without G6PD deficiency

Day 14	Coef	t value	[95% CI]	p value
Sex: base male	0			
Female	− 0.016	− 1.40	− 0.39–0.07	0.163
Age	0.012	2.29	0.00–0.02	0.023
G6PD status				
G6PDd ACT alone	− 0.44	− 1.19	− 1.17–0.29	0.234
G6PDn ACT + SLD-PQ	− 0.10	− 0.85	− 0.34–0.13	0.397
G6PDd ACT + SLD-PQ	− 0.45	− 1.61	− 1.01–0.10	0.108

Coef, mean Hb coefficient of variation estimated using a multiple linear regression model comparing the mean reduction in Hb concentration on day 14 in each group

G6PD-d: glucose-6-phosphate dehydrogenase deficiency, G6PD-n: normal glucose-6-phosphate dehydrogenase

## Abbreviations

ACT: Artemisinin-based combination therapy
AMM: Adjusted male median (AMM)
CI: Confidence interval
FMoH: Federal Ministry of Health
G6PD: Glucose-6-phosphate dehydrogenase
G6PDd: Glucose-6-phosphate dehydrogenase deficient
Hb: Haemoglobin
ICEMR: International Center of Excellence for Malaria Research
IQR: Interquartile Range
NIH: National Institute of Health
SD: Standard deviation
SLD-PQ: Single low-dose (0.25 mg/kg) primaquine
WHO: World Health Organization
